# Molecules Clarify a Cnidarian Life Cycle – The “Hydrozoan” *Microhydrula limopsicola* Is an Early Life Stage of the Staurozoan *Haliclystus antarcticus*

**DOI:** 10.1371/journal.pone.0010182

**Published:** 2010-04-14

**Authors:** Lucília S. Miranda, Allen G. Collins, Antonio C. Marques

**Affiliations:** 1 Departamento de Zoologia, Instituto de Biociências, Universidade de São Paulo, São Paulo, Brazil; 2 National Systematics Laboratory, National Marine Fisheries Service (NMFS), National Museum of Natural History, MRC-153, Smithsonian Institution, Washington, D. C., United States of America; Northeastern University, United States of America

## Abstract

**Background:**

Life cycles of medusozoan cnidarians vary widely, and have been difficult to document, especially in the most recently proposed class Staurozoa. However, molecular data can be a useful tool to elucidate medusozoan life cycles by tying together different life history stages.

**Methodology/Principal Findings:**

Genetic data from fast-evolving molecular markers (mitochondrial 16S, nuclear ITS1, and nuclear ITS2) show that animals that were presumed to be a hydrozoan, *Microhydrula limopsicola* (Limnomedusae, Microhydrulidae), are actually an early stage of the life cycle of the staurozoan *Haliclystus antarcticus* (Stauromedusae, Lucernariidae).

**Conclusions/Significance:**

Similarity between the haplotypes of three markers of *Microhydrula limopsicola* and *Haliclystus antarcticus* settles the identity of these taxa, expanding our understanding of the staurozoan life cycle, which was thought to be more straightforward and simple. A synthetic discussion of prior observations makes sense of the morphological, histological and behavioral similarities/congruence between *Microhydrula* and *Haliclystus*. The consequences are likely to be replicated in other medusozoan groups. For instance we hypothesize that other species of Microhydrulidae are likely to represent life stages of other species of Staurozoa.

## Introduction

Medusozoan (i.e., non-Anthozoan cnidarians) life cycles are highly complex and diverse, with combinations of planulae, benthic polyps (occasionally planktonic), creeping frustules, and/or pelagic medusae (occasionally benthic). As with other organisms displaying complex life cycles, documenting all the life history stages in medusozoan species is an enormous challenge. The usual approach has been to attempt to rear species through their various life stages in the laboratory. However, each life stage is adapted for different and often unknown conditions, making the task difficult, time consuming, and in many cases so far, impossible. Because the genome is the same in different life history stages of any given species, molecular data provide another tool that can help elucidate medusozoan life cycles by tying together different life stages.

While there is great variation in medusozoan life cycles, there exist some broad-scale patterns of congruence between life cycle differences and the origins of major medusozoan taxa [1], [2], suggesting that evolutionary changes in life cycle have sometimes corresponded to the establishment of distinct lineages. One of the most intriguing findings from these phylogenetic studies has been the hypothesis that the Stauromedusae (so-called stalked jellyfishes) form an early-diverging medusozoan clade that is separate from Scyphozoa (Coronatae and Discomedusae), within which Stauromedusae was traditionally classified [2], [3], [4], [5]. Because of its distinct origin and some putatively unique life history characteristics, Marques and Collins [2] established the class Staurozoa and noted that the finding raises important issues about the evolution of cnidarian development and life cycles.

The present view holds that the life cycle of staurozoans is relatively simple, consisting of a planula larva that attaches to the substrate and grows into a primary polyp, which subsequently undergoes an apical transformation into the adult form. Because the transformation to adult takes place without fission or budding, this development results in a mosaic individual, in which the structures of the oral part are similar to those of an adult medusa (particularly scyphozoans and cubozoans), whereas the basal part retains characteristics of the sessile polyp [6], [7]. However, knowledge of staurozoan development is based on a handful of observations on a small number of species. Studies about juvenile stauropolyp development include only *Haliclystus octoradiatus* (Lamarck, 1816) [6], [8] and two species of *Stylocoronella*, *S. riedli* Salvini-Plawen, 1966 and *S. variabilis* Salvini-Plawen, 1987 [7], [9]. Polyps of these latter species are interstitial and it is unknown whether or not this psammic condition is common in the group.

Based on analyses of nuclear genes coding for the small and large subunits of the ribosome (SSU or 18S and LSU or 28S, respectively), Collins and co-workers [10] suggested that the diminutive polyp form of the Antarctic species *Microhydrula limopsicola*, originally described by Jarms and Tiemann [11] in the class Hydrozoa (Trachylina, Limnomedusae), could be an unknown life stage of a species of Stauromedusae. The hypothesis was immersed in a broader analysis of the phylogeny and evolution of Trachylina, and many issues remain unattended: (1) To which staurozoan species should *M. limopsicola* be synonymized? (2) Which stage of the stauromedusan life cycle does it represent? (3) How can its morphology be interpreted in relation to what is known about staurozoans? and (4) What are the consequences of this unknown stage for our understanding of the biology and biogeography of staurozoans? The goal of this study is to address these questions, bringing new molecular and morphological evidence to this conundrum.

## Methods

**Figure 1:**
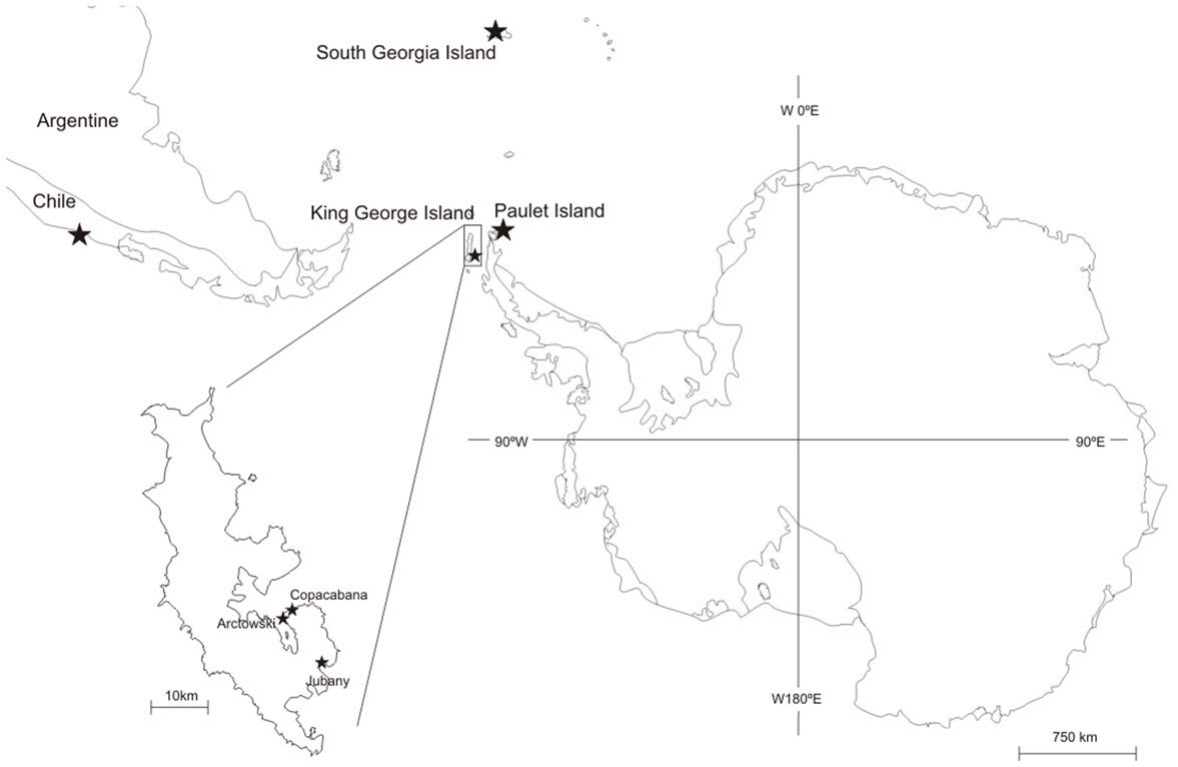
Map of Antarctica and southernmost part of Chile. Stars are records of *Haliclystus antarcticus*: South Georgia Island, Paulet Island, King George Island (Polish “Arctowski” Station, US “Copacabana” Refuge and Argentinean Antarctic Station “Jubany”) and Chile (Valdivia).

**Figure 2:**
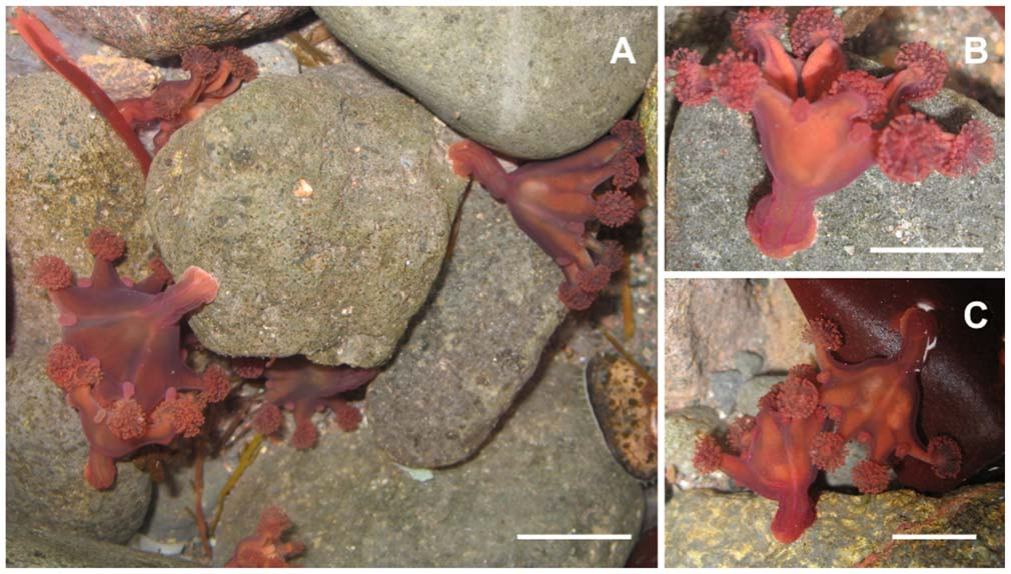
Living specimens of *Haliclystus antarcticus* in the field. A and B) Side view, attached to rock; C) Side view attached to rock and algae (Rhodophyta *Iridaea cordata*). Pictures from Morandini, AC. Scale = 1.2 cm.

We were provided with a few live polyps of *Microhydrula limopsicola* from a culture maintained by Gerhard Jarms at the Universität Hamburg. The culture, which has been maintained since December, 1991, derived from the original (and unique) sampling of this species on the shells of five specimens, 3–4 mm bivalves *Limopsis hirtella* (Rochebrune and Mabille, 1889) at 31 m deep in firm mud near the Argentine Antarctic Station “Jubany” (King George Island, South Shetland Islands 62°13.979'S 58°41.812'W; Figure 1, Table 1) [10], [11]. Specimens of *Haliclystus antarcticus* Pfeffer, 1889 from Antarctica (Figure 2) were collected manually during low tide (tide prediction between 0.2 and 0.4 m) on two beaches in the Admiralty Bay, King George Island, Antarctic Peninsula: (A) Pieter Lenie, Copacabana, North American Refuge, 62°10'S, 58°26'W; and (B) Shag Point, Arctowski, Polish Station, 62°10'S, 58°31'W (Figure 1, Table 1) and initially preserved in 80% ethanol [12]. Specimens of the Chilean *Haliclystus antarcticus*, originally (mis)identified as *H. auricula* (Rathke, 1806) [12], came from Los Molinos beach, Valdivia, southern Chile (39°47'S 73°20'W; Figure 1, Table 1), and were collected during low tide by C.J. Zagal [13], [14], [15] and J.P. Didier [12]. Tissue samples from the tentacle clusters were then dissected and preserved in pure ethanol stored at −20°C.

**Table 1:** Localities, GenBank codes (*sequences produced in this study) and number of specimens used in molecular analysis for each species and for each molecular marker.

Species	Locality	GenBank code		Number of specimens	Voucher
		16S	ITS1+ITS2		
*H. antarcticus*	Copacabana and Arctowski Sta (King George Island, Antarctica)	FJ874775*	FJ858787*	10	MZUSP 1558
*H. antarcticus*	Los Molinos (Valdivia, Chile)	AY845340	FJ874777*	10	MZUSP 1560
*“M. limopsicola”*	Jubany Sta (King George Island, Antarctica)	EU294003	FJ874779*	2 samples of the original culture	G. Jarms culture
*H. “sanjuanensis”*	San Juan Island (Washington, USA)	HM022151*AY845339	HM022145*HM022143*	11	USNM 1106935USNM 1073340
*H. “sanjuanensis”*	Franklin Point (California, USA)	HM022149*HM022150*	FJ874776*HM022144*	31	USNM 1106653USNM 1073341
*H. stejnegeri*	Muroran (Hokkaido, Japan)	HM022152*	HM022146*	1	USNM 1106655
*H. stejnegeri*	Akkeshi (Hokkaido, Japan)	HM022153*	HM022147*	1	KUNHM 002673-B
*H. tenuis*	Muroran (Hokkaido, Japan)	HM022154*	HM022148*	1	USNM 1106651
*D. africana*	False Bay (South Africa)	AY845341	HM022142*	1	none
*L. janetae*	East Pacific Rise (8°36.745N, 104°12.740W)	AY845342	FJ874778*	1	FMNH 10329

Sequences included in our analysis were derived for this study or have come from GenBank (Table 1). Fast evolving molecular markers (mitochondrial 16S, nuclear ITS1, and nuclear ITS2) were targeted for analysis. The markers were already adopted and proved to be efficient for the species level identification in Medusozoans (16S- [16], [17], [18]; ITS- [19]). DNA extraction was carried out with InstaGene (Bio-Rad). Genes were amplified using PCR, then purified with AMPure® (Agencourt®). PCR primers were CB1 (forward) and CB2 (reverse) [20] for mitochondrial DNA 16S; JFITS1-5f (forward) [21] and CAS28SB1d (reverse) [22] for nuclear ribosomal DNA (ITS1 and ITS2). DNA sequencing was made using the BigDye® Terminator v3.1 kit (Applied Biosystems) and the same primers for PCR, except by the use of ITS1-R (reverse) [23] for ITS1 and ITS2. The procedure was carried out on an ABI PRISM®3100 genetic analyzer (Hitachi). Samples of *M. limopsicola* and *H. antarcticus* were extracted and amplified at different times and at different laboratories (NMNH, USA and USP, Brazil, respectively), without risk of contamination. To confirm molecular data, the sequences of *M. limopsicola* (based on an independent DNA extraction from a second sampling of the original culture) and *H. antarcticus* (based on 10 individuals for each locality – King George Island, Antarctica and Valdivia, Chile – Table 1) were repeated at the same laboratories. Sequences of *M. limopsicola* were included in an analysis with mitochondrial 16S and nuclear ITS1 and ITS2 sequences of *H. antarcticus* (from Antarctica and Chile), *Haliclystus “sanjuanensis”* (*nomen nudum*) [24], *Haliclystus stejnegeri* Kishinouye, 1899, *Haliclystus tenuis* Kishinouye, 1910, *Depastromorpha africana* Carlgren, 1935, and *Lucernaria janetae* Collins and Daly, 2005 as an outgroup for rooting the topology (Table 1).

Contig sequences were edited in SEQUENCHER™ 4.6 (Gene Codes Corporation), aligned using BioEdit© “ClustalW Multiple Alignment” [25], resulting in three alignments: (1) mitochondrial 16S sequences, (2) ITS1+ITS2 sequences, and (3) combined 16S+ITS1+ITS2 sequences. Uncorrected pairwaise distances were calculated in Bioedit. Gaps were treated as missing data. Maximum Parsimony (MP) analyses were performed using branch and bound algorithm in PAUP 4.1 [26]. Maximum Likelihood (ML) analyses were performed using PALM (Phylogenetic Inference with Automatic Likelihood Model Selectors) [27]. The most appropriate model for each of the datasets was chosen by employing the Akaike information criterion (AIC). The model ‘GTR+I+G’ was applied to 16S, ‘TIMef’ to ITS1+ITS2 and ‘GTR+G’ to combined data (16S+ITS1+ITS2). Branch support was estimated by bootstrapping [28] with 1000 replicates for the MP (PAUP 4.1) and ML (PALM) analyses.

## Results

**Figure 3:**
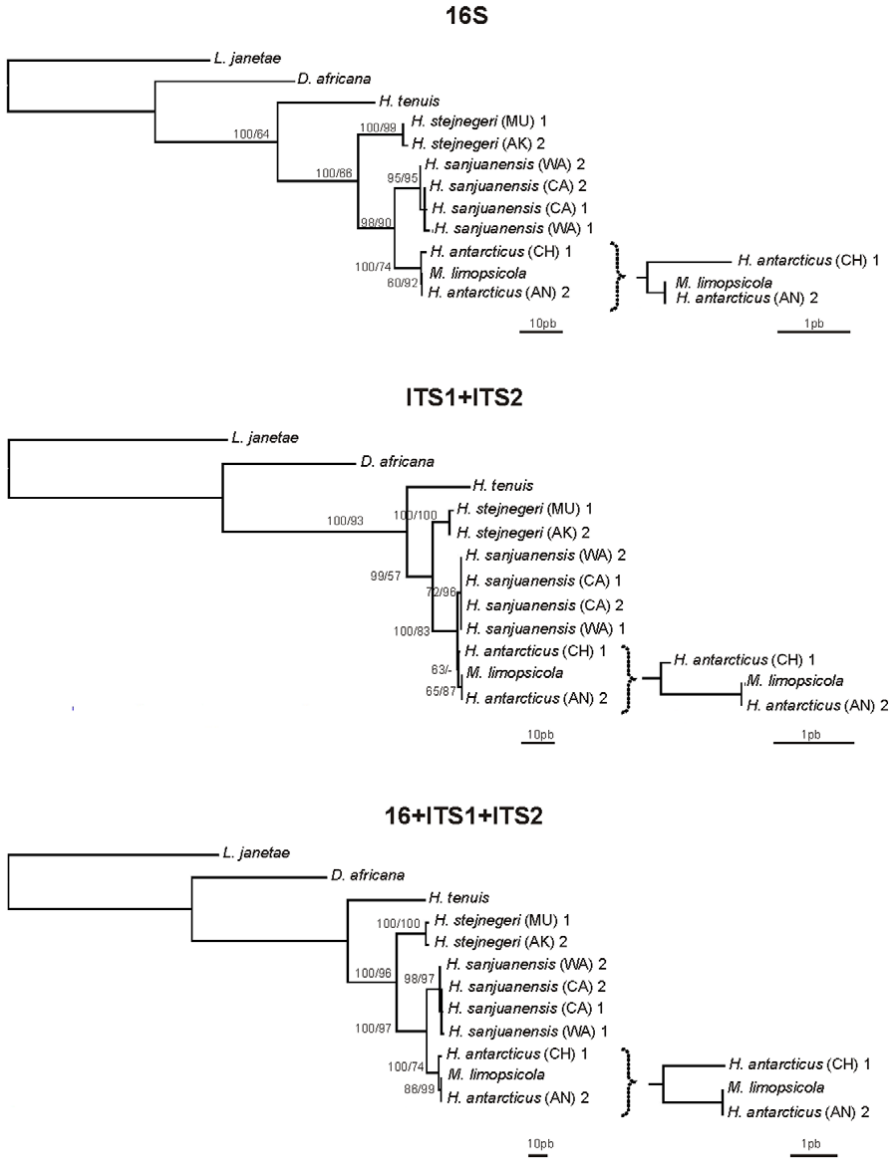
Phylogenetic hypothesis (MP) based on mitochondrial 16S, nuclear ITS1+ITS2 and combined data. AN (King George Island, Antarctica), AK (Akkeshi, Hokkaido, Japan), CA (Franklin Point, California, USA), CH (Valdivia, Chile), MU (Muroran, Hokkaido, Japan), WA (San Juan Island, Washington, USA).“1” and “2” refers to the different haplotypes found for each species. Bootstrap indices under both MP and ML (respectively) at each node. Topologies are congruent under MP and ML analysis.

The MP and ML topologies are identical for 16S and combined data. The ML topology for ITS1+ITS2 is congruent with MP topology, however less resolved (Figure 3). The cladograms show that *M. limopsicola* from Antarctica falls within *H. antarcticus*, and in fact, has no differences from *H. antarcticus* of Antarctica. *Haliclystus antarcticus* from Chile forms a clade with these specimens and is only slightly diverged from them.

All ten sequenced specimens of *H. antarcticus* from Antarctica possess a unique haplotype for the three markers, and this is identical (for 16S, ITS1 and ITS2 - Table 2) to the haplotype found for *M. limopsicola* and slightly different (distance of 0.40–0.77%, depending on the marker - Table 2) from the haplotype of the *H. antarcticus* from Chile. Similar to *H. antarcticus* from Antarctica, the population from Chile (n = 10) has no genetic variation for the studied molecular markers, presenting a unique haplotype (Tables 1 and 2). Nucleotide differences among 16S, ITS1 and ITS2 sequences from other species of Stauromedusae (*H. “sanjuanensis”*, *H. stejnegeri*, *H. tenuis*, *D. africana* and *L. janetae*) are higher (3.75–33.78% for 16S; 1.22–50.28% for ITS1; and 0.94–61.15% for ITS2; Table 2 - phylograms in Figure 3). Species of stauromedusae, for which we have more than one haplotype (*H. antarcticus*, *H. “sanjuanensis”* and *H. stejnegeri*) show that the intraspecific difference is between 0.19–0.77% for 16S, 0.00–0.40% for ITS1 and 0.00–0.47% for ITS2 (Table 3).

**Table 2:** DNA distance matrix between *Haliclystus antarcticus* from Antarctica and: “*Microhydrula limopsicola*”, *Haliclystus antarcticus* from Chile, *Haliclystus* “*sanjuanensis*”, *Haliclystus stejnegeri*, *Haliclystus tenuis*, *Depastromorpha africana* (family Depastridae) and *Lucernaria janetae* (family Lucernariidae).

	*H. antarcticus* (King George Island, Antarctica)		
	16S	ITS1	ITS2
***“M. limopsicola”*** ** (King George Island, Antarctica)**	0.00%	0.00%	0.00%
***H. antarcticus*** ** (Valdivia, Chile)**	0.77%	0.40%	0.47%
***H. “sanjuanensis”*** ** (San Juan Island, Washington) Haplotype 1**	3.75%	1.22%	0.94%
***H. “sanjuanensis”*** ** (San Juan Island, Washington) Haplotype 2**	3.75%	1.22%	0.94%
***H. “sanjuanensis”*** ** (Franklin Point, California) Haplotype 1**	3.75%	1.22%	0.94%
***H. “sanjuanensis”*** ** (Franklin Point, California) Haplotype 2**	3.96%	1.22%	1.41%
***H. stejnegeri*** ** (Muroran, Japan) Haplotype 1**	7.16%	2.05%	6.00%
***H. stejnegeri*** ** (Akkeshi, Japan) Haplotype 2**	7.37%	2.47%	6.00%
***H. tenuis***	16.41%	7.95%	13.35%
***D. Africana***	22.06%	20.74%	26.24%
***L. janetae***	33.78%	50.38%	61.15%

**Table 3:** Intraspecific variation for three species of Staurozoa in 16S, ITS1 and ITS2, highlighting the number of specimens, the number of haplotypes found and the range of divergence of each molecular marker; the linear distance refers to the distance between populations.

Species		*H. antarcticus*	*H. “sanjuanensis”*	*H. stejnegeri*
Locality		King George Island (Antarctica) and Valdivia (Chile)	Washington and California (Pacific Coast, USA)	Akkeshi and Muroran (Hokkaido, Japan)
**Linear distance**		2,700 km	1,270 km	330 km
**Population**		2	2	2
**16S**	Specimens	20	6	2
	Haplotypes	2	4	2
	Divergence	0.77%	0.19–0.39%	0.19%
**ITS1**	Specimens	20	4	2
	Haplotypes	2	1	2
	Divergence	0.40%	-	0.40%
**ITS2**	Specimens	20	4	2
	Haplotypes	2	2	1
	Divergence	0.47%	0.46%	-

## Discussion

### Microhydrula limopsicola is Haliclystus antarcticus

Collins and co-workers [10] did not formally establish a synonym for *Microhydrula limopsicola*. However, a very close relationship of *M. limopsicola* with *H. octoradiatus* and also with “the species of *Haliclystus* from southern Chile reported on by Zagal (2004)” was noted, although the latter was not formally included in their analysis [10]. Our analyses are obviously constrained by the non-availability of other cultures or samples of *M. limopsicola*, which as far as we know has only been observed by Jarms and Tiemann [11], but our results are based on the most complete data available at this time. With increased taxon sampling and data from fast-evolving markers, we conclude that *M. limopsicola* is actually *H. antarcticus*. Several points support this conclusion.

First, we document 100% identity of three fast-evolving markers of *Microhydrula limopsicola* with those of *H. antarcticus* from Antarctica, all of which differ slightly from conspecific samples of *H. antarcticus* from Chile. While intraspecific variation of these genetic markers is not very well known for species of stauromedusae, available data indicate that some intraspecific genetic variation exists, and that it is smaller than observed interspecific variation (compare Table 3 with Table 2). This would suggest that identity in these genetic markers can only happen if the samples are taken from the same species. Finally, one might question whether there are other Antarctic species of *Haliclystus* that could confound our identification of *Microhydrula limopsicola* as *H. antarcticus*. There is one additional species known from the southern hemisphere, *Haliclystus kerguelensis* Vanhöffen, 1908 from Kerguelen Island, southern Indian Ocean, but this species is readily differentiated from *H. antarcticus* by its morphology [12] and thus should have a different genetic signature; no specimens of *H. kerguelensis* suitable for genetic study were available to us.

With the remainder of the discussion, we synthesize the relevant historical literature to address the implications of our identification of *M. limopsicola* as *H. antarcticus* on taxonomy, morphology, and life history of Stauromedusae.

### Taxonomy

The implications of this synonymy for the family Microhydrulidae were only briefly touched upon by Collins and co-workers [10]. The family Microhydrulidae (Hydrozoa, Limnomedusae) encompassed three species in two genera: *Microhydrula pontica* Valkanov, 1965, *M. limopsicola* and *Rhaptapagis cantacuzenei* Bouillon and Deroux, 1967. One is now clearly established as a stauromedusa. It remains to be explicitly tested whether *M. pontica* and *R. cantacuzenei* are also early stages of the life cycle of local species of Stauromedusae, but we think this is likely to be the case, since similarities between these species and the preserved larvae of stauromedusae have been previously recorded [29]. We note that *M. pontica* and *R. cantacuzenei* have been found in abundance living in marine surface biofilms at the Station Biologique in Roscoff, France, on the English Channel, in the same vicinity where several species of Staurozoa including *Haliclystus auricula*, *H. octoradiatus*, *Depastrum cyathiforme* (M. Sars, 1846), *Lucernariopsis campanulata* (Lamouroux, 1815) and *Craterolophus convolvulus* (Johnston, 1835) [6], [29], [30], [31] have also been found. Of course, additional data, particularly genetic data, from other species of Microhydrulidae are necessary to test our hypothesis that these species represent stages in the development of species of Stauromedusae.

### Morphology

Medusozoan plesiomorphies (e.g., primitive widespread cnidarian characters such as the presence of microbasic euryteles nematocysts), homoplasies (e.g., presence of convergent morphological characters such as periderm and life history characters such as asexual frustules), and the very simple morphology (small solitary hydroids without tentacles and sexual stage [11], [29]) of *M. limopsicola* evidently worked as obstacles in correctly identifying *M. limopsicola* when it was discovered. As a result, *M. limopsicola* bears a closely resemblance to the very simple hydropolyps such as those of *Craspedacusta* and *Monobrachium* [11]. No doubt the scarce literature on early stages of staurozoans made it difficult to establish reliable comparisons among taxa. Even though dissimilarities are evident [10], there are several morphological similarities between *M. limopsicola* and Staurozoa.

**Figure 4:**
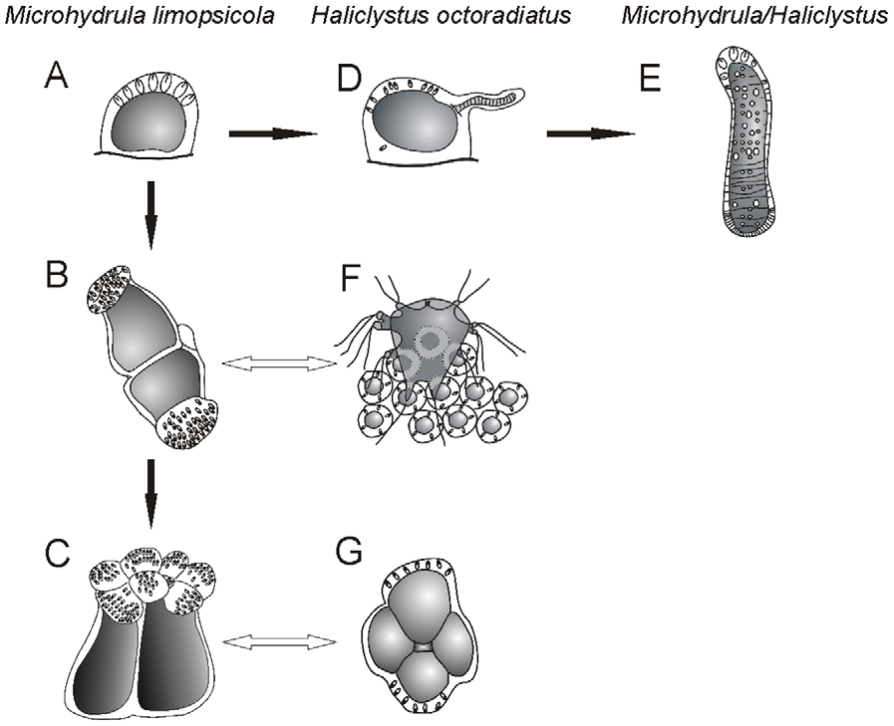
Comparisons between the *M. limopsicola* polyp and the *H. octoradiatus* settled planula. *(A–C) different stages of M. limopsicola* [11]: A) newly settled “polyp”; B) closely attached “polyps”, with expansions provided with nematocysts; C) later stage, with a cauliflower-shaped head. *(D–E) process of frustulation observed in both species*: D) planula of *H. octoradiatus* producing lateral protuberances, which become frustules [6]; E) frustules [11]. *(F–G) possible correspondences of stages of both species*: F) a group of *H. octoradiatus* larvae, capturing a nauplius [6]; G) superior view of a settled planula of *H. octoradiatus* at an advanced stage, showing four lobes [8].The hemispherical shape and the production of frustules (A, D, E) are similar in settled planulae of *H. octoradiatus* and “polyps” of *M. limopsicola*. The same gregarious behavior to feeding was observed in both species (B, F). At a more advanced stage, the larva of *H. octoradiatus* presents four lobes (G), that might be associated with the cauliflower structure seen in later stages of *M. limopsicola* (C), which possibly is an aggregation of more than one individual. Figures modified from [6], [8], [11].

First, the hemispherical shape of the settled planulae of *Haliclystus* octoradiatus [6] is similar to the general shape of Microhydrulidae “polyps” [11] (Figure 4). Moreover, both lack mouth and a permanent gastrovascular cavity necessitating intracellular digestion [6], [11], [29]. A further similarity is the production of frustules [6], [11]. More specifically, the settled, rounded up planula of *H. octoradiatus* produces lateral protuberances, which become buds for the process of frustulation [6], similar to the “young polyps of *M. limopsicola*”, which can also produce frustules [11] (Figure 4). Likewise, the frustules of Microhydrulidae are formed from a lateral budding of the body [29]. The planulae of *H. octoradiatus* can also have small pronounced expansions, more or less regular, not related to the process of frustulation. These protuberances, which are provided with numerous nematocysts, result from a local thickening of the ectoderm and seem to play an important role in prey capture prior to the development of tentacles [6]. Homology between the protuberances, found in *H. octoradiatus* planulae larvae and the similar morphology in *M. limopsicola* is not clear. One possible interpretation is that these expansions correspond to the cauliflower structure bearing numerous nematocysts found at one end of “elder” individuals of *M. limopsicola* [11]. However, the cauliflower structure can also be a simple result of a strong aggregation of larvae (see below) [6] since at a more advanced stage, the larva of *H. octoradiatus* increases considerably in size, and its contour, previously more or less rounded, develop four lobules [8] in a similar arrangement to the cauliflower structure (Figure 4). Another interpretation is that the development of *H. antarcticus* differs from that of *H. octoradiatus*, with the cauliflower structure corresponding to the early development of the primary tentacles. Recently metamorphosed individuals of *Haliclystus borealis* Uchida, 1933, *H. stejnegeri*, and *H. auricula*, as well as their juvenile medusae stages, have eight primary tentacles [32], which might be correlated to these cauliflower structures.

Histological similarities between *M. limopsicola* and *H. antarcticus* are also of note. The “polyps” of *M. limopsicola* are attached to the substratum with a slightly widened base, whose ectoderm produces a flat thin fibrous periderm plate [11]. Stalks of adult individuals of the genus *Haliclystus* also present fibrillar components at the attachment sites [33]. In addition, the endoderm of *M. limopsicola* and of the settled planula of *H. octoradiatus* is composed by vacuolar cylindrical cells, which touch at the terminal end, without leaving any space [6], [11].

One feature remarked by Jarms and Tiemann [11] and Collins and co-workers [10] is the cnidome, with microbasic euryteles being present in *M. limopsicola*. This nematocyst type is a common feature of *H. antarcticus* [12], [34], but microbasic euryteles are plesiomorphic for Staurozoa [3], [32], [35], [36], [37], [38], [39], [40] and not particularly useful for staurozoan taxonomy, besides its ubiquitous presence in other medusozoan groups (see data matrix in [2]). Adults of *H. antarcticus* also possess isorhizas [12], [34], which were not recorded for *M. limopsicola* [11]. Accepting the identity of *M. limopsicola* with *H. antarcticus* suggests that the cnidome of this species varies ontogenetically [38], [41], [42]. Indeed, nematocysts of the creeping planula larvae of *Haliclystus salpinx* Clark, 1863 are different from the adults of the same species [43]. The planula larval stage of *H. salpinx* also has only microbasic euryteles, whereas the adults have both microbasic euryteles and isorhizas [43].

### Life cycle inference

**Figure 5:**
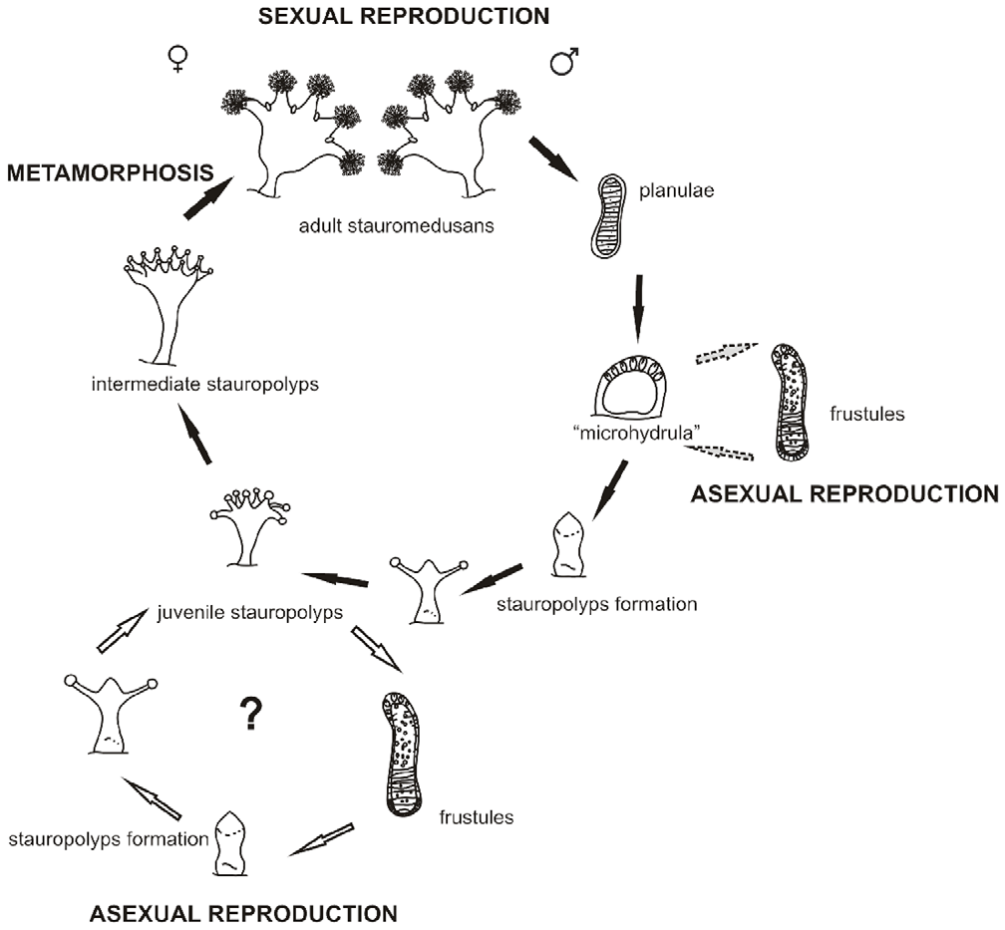
Putative scheme of the life cycle of *H. antarcticus*, including the “microhydrula” phase. The main life cycle was based on [6], for *H. octoradiatus*. Stauropolyp stage and its ability to create frustules (white arrows) are hypothesized based on observations of *Stylocoronella* [7]. Dotted gray arrows corresponding to the “microhydrula” stage, derived from this study. Figures modified from [6], [7], [11].

Based on our finding that *M. limopsicola* is synonymous with *H. antarcticus* we propose that *M. limopsicola* is an early life cycle stage of *H. antarcticus* (Figure 5). Thus far, early stages of the life cycle of *H. antarcticus* have never been recorded. In fact, few staurozoan pre-adult stages are known. Only the creeping benthic planula stage of *H. “sanjuanensis”* (misidentified as *H. stejnegeri*) and *H. salpinx* [43], [44], the post-metamorphosis stages of some stauromedusae [32] and the complete development of *H. octoradiatus*, *S. riedli* and *S. variabilis* [6], [7] have been documented. Therefore, it is presently impossible to assert that the Microhydrula stage is present in all species of Staurozoa. Similarly, it is unclear how widespread the presence of frustule stages is in other stauromedusae. Nevertheless, we suggest that the study of sediments and potential associations with overlooked substrata (e.g., bivalves) is likely to reveal a hidden diversity of life cycle stages and strategies in Staurozoa, which are generally assumed to live only on rocks and macrophytes.

The non-sexually-reproductive “microhydrula” stage most likely occurs before the development of the stauropolyp and its apical metamorphosis into an adult medusa in the life cycle. We hypothesize that this stage occurs right after the settlement of the planula larvae (or sometime after the settlement of the frustule), adding a stage (Figure 5) to the hypothetical life cycle proposed for staurozoans, since the best known life cycle of a staurozoan, the odd psammic *Stylocoronella* spp., apparently does not contain a “microhydrula” stage [7]. Settlement of planulae occurs in groups of 3–20 individuals in *H. octoradiatus* [6]. Experimental procedures restricting larval aggregation of *H. octoradiatus* to groups of 1–3 larvae demonstrated that none of these larvae were successful in undertaking further development, probably due to not being able to capture sufficient food when growing in small aggregations [6]. The same gregarious behavior has been described for “adults” of Microhydrulidae, also hypothesized to enhance efficiency in prey capture [11], [29] (Figure 4). Furthermore, similar to planula settlement in *H. octoradiatus*, the frustules of Microhydrulidae attach to the substrate by adhesion of a portion of the surface lacking nematocysts [6], [11], [29].

It is important to note, however, that the juvenile stauropolyps of *S. riedli* and *S. variabilis* can produce frustules by budding of the long filiform tentacles [7]. Such a polyp has not been described for any other staurozoan. Asexual reproduction via frustulation found in the “microhydrula” stage (and possibly also in the polyp, since cnidarians demonstrate different kinds of budding [45], [46]) would increase the potential of large populations in isolated areas of a larger fragmented seascape, which is consistent with the patchy distribution of *H. antarcticus*, for example. In fact, 130 specimens of *H. antarcticus* were found in an area of ca. 150m^2^ in Copacabana, King George Island, Antarctica [12], and ca. 385 individuals/m^2^ in Valdivia, Chile [14]. Further, intense asexual reproduction would lead to low genetic diversity. This is consistent with our finding of just single 16S and ITS haplotypes (Table 2) in each sampled *H. antarcticus* population (Antarctica and Chile).

Ecological constraints of the “microhydrula” stage may restrict the distribution of *H. antarcticus*. *Microhydrula limopsicola* was described living attached to the upper valve of the very small, subtidal lamellibranch bivalve *Limopsis hirtella* on King George Island [11]. Jarms and Tiemann [11] suggested that the association between the clam *Limopsis hirtella* and the hydroid *Microhydrula limopsicola* is to be regarded as “highly specific”. Jarms and Tiemann have kept *M. limopsicola* alive on glass for nearly 20 years and, to our knowledge, no development other than asexual frustulation has been observed [11]. Since *M. limopsicola* can be kept alive in the lab, the association between *M. limopsicola* and *L. hirtella* may be regarded as a coincidence of co-distribution. However, the absence of further development in the lab leads us to believe that *L. hirtella* provides vital cues enabling further development of *M. limopsicola*. Such relationships among epibiont and host are not uncommon in marine ecosystems [47]. *Limopsis hirtella* is spread across the Magellanic Province, the Falkland Islands and the western part of the Antarctic [48]. Coincidentally, this is the area where *H. antarcticus* is recorded: Antarctica Peninsula [12], [34], [49], [50] and southern South America (Figure 1) [12], [13], [14], [15], [51].

### Conclusions

Documenting medusozoan life cycles is an enormous challenge. In this work we show that molecular data can be a useful tool to identify an unknown life cycle stage by tying together different life history stages with the same haplotype and from it derive a hypothesis about the life history of the species. Similarity between the haplotypes of three markers of *Microhydrula limopsicola* and *Haliclystus antarcticus* settles the identity of these taxa, expanding our understanding of the staurozoan life cycle, which was thought to be more straightforward and simple. Frustulation was recorded for *Haliclystus* for the second time, in a different life stage from the one recorded for *Stylocoronella*. This knowledge sheds light on morphological, biogeographical, and evolutionary issues, mainly because *Haliclystus* is the most diverse genus in Staurozoa. However molecular analysis will not replace additional investigations. Continued exploration of the meiofauna and integrated analysis encompassing morphology, ecology, molecules, life cycles and biology will be needed to solve outstanding evolutionary and biogeographical questions like those addressed here.
